# Antibody-drug conjugates in lung cancer: scientific breakthroughs and future directions in clinical trials

**DOI:** 10.1097/JS9.0000000000002718

**Published:** 2025-06-12

**Authors:** Qing-Dong Zhu, Chang Song, Chun-Yan Zhao, Hang-Biao Qiang, Ren-Hao Liu, Chun-Ming Gong, Qiu-Qing Tan, Chang-Yue Jiang, Mei Yu

**Affiliations:** a Department of Tuberculosis, The Fourth People’s Hospital of Nanning, Nanning, Guangxi, China; b Guangxi Medical University, Nanning, Guangxi, China; c Department of Pharmacy, The Fourth People’s Hospital of Nanning, Nanning, Guangxi, China; d Department of Anesthesiology, The Fourth People’s Hospital of Nanning, Nanning, Guangxi, China

Lung cancer remains the leading cause of cancer-related mortality worldwide, prompting the urgent need for innovative therapeutic approaches. Among emerging modalities, antibody-drug conjugates (ADCs) have demonstrated significant potential in lung cancer treatment due to their precision delivery mechanisms. By coupling monoclonal antibodies with highly potent cytotoxic agents, ADCs selectively target and eliminate tumor cells while minimizing damage on healthy tissues^[1]^. This design overcomes key limitations of traditional chemotherapy and provides new therapeutic options, especially in drug-resistant cases. With advancements in molecular biology and drug delivery platforms, ADCs have achieved remarkable clinical breakthroughs in both non-small cell lung cancer (NSCLC) and small cell lung cancer (SCLC). Numerous clinical trials have confirmed potent anti-tumor activity, particularly with ADCs targeting HER2, TROP2, and c-Met, confirming both their efficacy and safety^[2,3]^. Despite this progress, challenges remain, including optimizing target selection, understanding resistance mechanisms, and managing adverse effects. This article systematically reviews recent developments in lung cancer ADC clinical trials and discusses future research directions to support broader clinical translation and integration into precision oncology.

We performed a comprehensive analysis of lung cancer ADC trials using the INFORMA Pharma Database (https://pharma.id.informa.com/), employing composite search terms encompassing: [(Disease is Oncology: Lung, Non-Small Cell) OR (Disease is Oncology: Lung, Small Cell)] AND (Drug Type is Biological > Protein > Antibody > Antibody-drug conjugate). A double-blind screening process was conducted independently by two researchers, with disagreements resolved by a senior researcher. A total of 510 clinical trials were included, and data were analyzed across multiple dimensions, including spatiotemporal distribution characteristics, funding models, molecular targets and mechanisms, and clinical trial phase characteristics. This approach revealed both the evolving trajectory and key technological advances in ADC-based lung cancer therapy. All aspects of this study, including design, data analysis, and conclusion derivation, were exclusively and independently executed by the researchers without the use of any artificial intelligence (AI) tools. Recognizing the importance of transparency in AI-related research, we adhered to the TITAN guidelines proposed by Agha *et al*, which provide detailed guidance for the transparent reporting of AI research^[4]^.

**HIGHLIGHTS**

- Analysis of 510 trials reveals rapid NSCLC/SCLC ADC development, led by China, the United States, and Europe, with Phase III trials surging in 2024.
- TROP2/HER2 ADCs improve survival in NSCLC; emerging targets (DLL3, MET) and unresolved resistance/toxicity issues are discussed.
- Regulatory frameworks (21st Century Cures Act, NMPA guidelines) and innovations (dual-epitope ADCs) accelerate clinical translation.

Since 2001, ADC clinical trials of lung cancer have steadily increased, with a marked rise in Phase I and II trials, and a significant expansion of Phase III trials in 2024 (Fig. 1A). However, Phase IV trials remain limited (Fig. 1B), highlighting a bottleneck in post-marketing validation. Globally, China, the United States, and Europe dominate the ADC clinical trial landscape, with single-center studies still prevailing (Fig. 1C and D). As global collaboration strengthens, multicenter and cross-regional clinical trial models are expected to become more prevalent. Pharmaceutical companies are the primary sponsors of lung cancer ADC trials. AbbVie (43 trials), Daiichi Sankyo (33 trials), and Pfizer (25 trials) lead globally, while Chinese companies such as Sichuan Baili Pharmaceutical (23 trials) and Jiangsu Hengrui Pharmaceuticals (19 trials) are also heavily involved (Fig. 1E and F).

**Figure 1.**
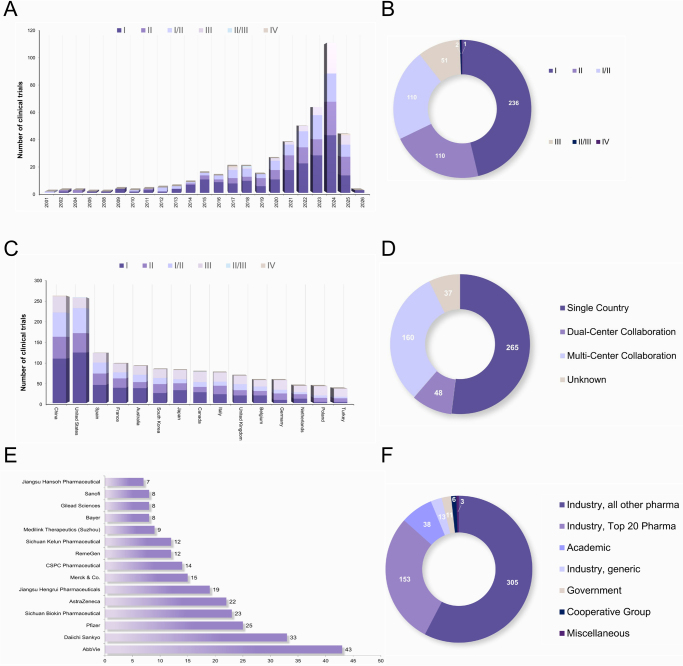
Overview of clinical trials for lung cancer ADC. (A) Annual trial distribution. (B) Distribution of trial phases. (C) Distribution of the top 15 countries conducting clinical trials for lung cancer ADC. (D) Collaborative landscape of clinical trials for lung cancer ADC. (E) Distribution of the top 15 sponsors for clinical trials of lung cancer ADC. (F) Types of funding organizations supporting clinical trials for lung cancer ADC.

Current clinical trial data hotspots are focused on the human epidermal growth factor receptor (HER) family and trophoblast cell surface antigen (TROP2) (Fig. 2). TROP2, a transmembrane glycoprotein involved in tumor proliferation and metastasis, is highly expressed in various malignant tumors^[5]^. Among the most extensively studied ADCs targeting TROP2 are datopotamab deruxtecan (Dato-DXd), sacituzumab tirumotecan (Sac-TMT), and sacituzumab govitecan (SG). Dato-DXd demonstrates enhanced plasma stability via engineered antibody structure^[6]^. In a Phase III trial, it improved median progression-free survival (PFS) to 4.4 months (95% CI, 4.2–5.6) compared to 3.7 months (95% CI, 2.9–4.2) with docetaxel in previously treated metastatic NSCLC^[7]^. Sac-TMT has shown efficacy in advanced NSCLC, regardless of EGFR mutations status^[8]^. SG, comprising an anti-TROP2 antibody conjugated to the topoisomerase I inhibitor, SN-38 (the active metabolite of irinotecan), showed a 1.3-month overall survival (OS) benefit over docetaxel (11.1 vs. 9.8 months, HR = 0.84), with an even greater benefit (3.5 months) in the anti-PD-L1 non-responsive subgroup (HR = 0.75). SG also demonstrated superior safety (treatment-related discontinuation: 6.8% vs. 14.2%; fatal toxicity: 1.4% vs. 1.0%)^[9]^.

**Figure 2.**
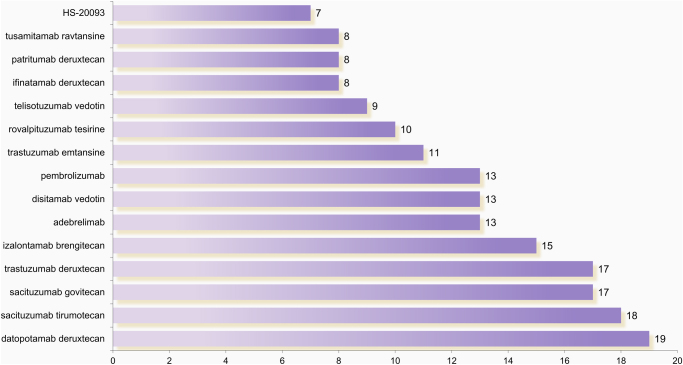
Distribution of the top 15 most common drugs in clinical trials for lung cancer ADC.

In HER2-overexpressing NSCLC, trastuzumab deruxtecan has delivered promising efficacy and safety^[10]^. Emerging targets such as Delta-like ligand 3 (DLL3) have garnered interest. Rovalpituzumab tesirine (Rova-T), a DLL3-targeting ADC, initially showed encouraging Phase II results in relapsed/refractory SCLC (ORR:18%, and median OS: 5.6 months). However, in the Phase III TAHOE trial due to inferior OS (OS: 6.3 vs. 8.6 months; HR = 1.46) and higher toxicity, including interstitial lung disease^[11]^. Technologically innovation is driving ADC evolution through dual-epitope constructs (e.g., izalontamab brengitecan) and novel payloads. ADCs targeting resistance pathways, such as telisotuzumab vedotin against the MET proto-oncogene, are entering clinical validation. Nevertheless, challenges remain, including tumor heterogeneity, target variability, and toxicity control. Future research must integrate refined biomarker screening and pharmacokinetic optimization to fully realize ADCs’ potential in precision lung cancer therapy.

Globally, regulatory frameworks are advancing to accelerate ADC innovation. In the United States, the 21st Century Cures Act facilitates expedited ADC approvals, including priority review for pivotal trials such as TROPION-Lung study led by AstraZeneca. The European Union supports public–private partnerships through the Innovative Medicines Initiative. In China, the Major New Drug Innovation Program prioritizes ADC development. In 2023, the National Medical Products Administration (NMPA) released the Technical Guidelines for Clinical Development of Anti-Tumor Antibody-Drug Conjugates, standardizing biomarker integration and dose optimization. Japan’s Sakigake Review System provides fast-track designation, reducing the standard review period from 12 months to 6 months. These coordinated policies have fostered rapid expansion in lung cancer ADC trials and created a global triad of regulatory innovation, financial support, and industrial collaboration. In summary, ADCs have provided therapeutic possibilities in lung cancer by integrating target innovation, payload optimization, and immune-based synergy. Future priorities include elucidating resistance mechanisms, enhancing toxicity control, and developing dynamic biomarker systems. Integration of multi-omics strategies and global trial networks will accelerate progress toward personalized, effective lung cancer therapies, ultimately improving patient survival and quality of life.

Based on data from 510 ADC clinical trials in lung cancer, this study provides a comprehensive analysis of spatiotemporal trends, funding models, molecular targets, and trial phases. Findings demonstrate the pivotal roles of TROP-2, HER2, and DLL3 in driving ADC development. By evaluating representative agents including SG, Trastuzumab Deruxtecan, and Rova-T, this study provides a robust theoretical foundation for future clinical application. These insights provide both theoretical and practical guidance for optimizing ADC strategies, refining treatment protocols, and advancing the implementation of personalized medicine in lung cancer care. The conclusions hold substantial academic and clinical value in guiding the ongoing innovation and translation of ADC technology.

## Data Availability

The datasets generated and analyzed during the current study are available in the INFORMA database (https://pharma.id.informa.com/).
